# Emodin Enhances Rosiglitazone’s Therapeutic Profile by Dual Modulation of SREBP1-Mediated Adipogenesis and PPARγ-Driven Thermogenesis

**DOI:** 10.3390/ph18121810

**Published:** 2025-11-27

**Authors:** Meng Li, Yi-Rong Wang, Xue Wang, Xiao-Li Xiao, Yun-Hong Sun, Sheng-An Zhang, Yan-Qi Dang, Kai Wang, Wen-Jun Zhou

**Affiliations:** 1 Institute of Digestive Diseases, Shanghai University of Traditional Chinese Medicine, Shanghai 200032, China; mengli@ntu.edu.sg (M.L.); wangyirong404@163.com (Y.-R.W.); wx130218@163.com (X.W.); xxl12zy@126.com (X.-L.X.); syh_010122@163.com (Y.-H.S.); zhangshengan@hotmail.com (S.-A.Z.); dangyanqi9022@126.com (Y.-Q.D.); 2 State Key Laboratory of Integration and Innovation of Classical Formula and Modern Chinese Medicine, Shanghai 201203, China; 3 School of Biological Sciences, Nanyang Technological University, Singapore 637551, Singapore; 4 CAS Engineering Laboratory for Nutrition, Shanghai Institute of Nutrition and Health, University of Chinese Academy of Sciences, Chinese Academy of Sciences, Shanghai 200031, China; 5 School of Public Health, Shanghai University of Traditional Chinese Medicine, Shanghai 201203, China; 6 Experiment Center for Science and Technology, Shanghai University of Traditional Chinese Medicine, Shanghai 201203, China; jeffrey_wangkai@hotmail.com

**Keywords:** emodin, rosiglitazone, PPARγ, type 2 diabetes, obesity, adipogenesis, thermogenesis, insulin sensitivity

## Abstract

**Background/Objectives:** Rosiglitazone (RSG), a potent PPARγ agonist for type 2 diabetes mellitus (T2DM), induces adverse adipogenic effects that limit clinical use. We investigated whether emodin (1,3,8-trihydroxy-6-methylanthraquinone, EMO), a natural anthraquinone, mitigates RSG-induced complications while enhancing its insulin-sensitizing benefits in severe obesity. **Methods:** Male *ob*/*ob* mice with established obesity and diabetes were treated for 4 weeks with RSG (10 mg kg^−1^ day^−1^), EMO (200 or 400 mg kg^−1^ day^−1^) or their combination. Metabolic profiling, organ function, and adipose histology were analyzed. RNA sequencing and mechanistic studies (Western blot, RT-qPCR, luciferase assays) in inguinal subcutaneous adipose tissue (iSAT), epididymal white adipose tissue (eWAT), and 3T3-L1 adipocytes were used to define EMO’s actions. **Results:** EMO co-treatment dose-dependently reduced RSG-induced weight gain, visceral adiposity (iSAT and eWAT mass, *p* < 0.05), and ectopic lipid deposition while ameliorating hepatorenal dysfunction. EMO synergistically enhanced RSG’s glucose-lowering effects. Mechanistically, EMO suppressed sterol regulatory element-binding protein 1 (SREBP1)-mediated lipogenesis (*Srebp1*, *Acc*, *Fasn*, *Scd1*; *p* < 0.05) and enhanced PPARγ-peroxisome proliferator-activated receptor gamma coactivator-1α (PGC-1α)-driven thermogenesis via enhanced PPARγ transactivation and nuclear translocation. Thermogenic genes (*Ucp1*, *Ppargc1a*, *Cidea*; *p* < 0.05) were upregulated, with maximal uncoupling protein 1 (UCP1) induction in iSAT at 400 mg/kg EMO. **Conclusions:** EMO selectively enhances RSG’s glycemic benefits while attenuating its adipogenic effects in severe obesity by dual PPARγ modulation-inhibiting adipogenic pathways while amplifying thermogenesis. This strategy mitigates RSG’s adverse effects while improving insulin sensitivity, supporting the potential of EMO as a PPARγ adjunct therapy.

## 1. Introduction

T2DM affects over 500 million adults globally, and its trajectory is still accelerating. The disease is driven by obesity-related insulin resistance and progressive β-cell dysfunction, creating a therapeutic dilemma: each drug class improves one metabolic axis while worsening another [1,2,3]. This metabolic dysfunction frequently coexists with and exacerbates conditions such as metabolic dysfunction-associated steatotic liver disease (MASLD) and dyslipidemia, creating a complex therapeutic landscape where managing one aspect often negatively impacts another. Current pharmacotherapies, while effective in certain domains, are hampered by significant adverse effects. Insulin sensitizers like thiazolidinediones (TZDs), secretagogues, and sodium-glucose cotransporter-2 (SGLT2) inhibitors can cause weight gain, hepatotoxicity, fluid retention, and hypoglycemia, limiting their long-term utility and patient compliance [4,5].

RSG, a potent and selective agonist for the nuclear receptor PPARγ, exemplifies this therapeutic dilemma. PPARγ activation by RSG effectively improves insulin sensitivity, primarily by reprogramming WAT to enhance lipid storage capacity, reduce lipotoxicity, and improve adipokine secretion [6]. However, this very mechanism underlies its major drawback: the concomitant activation of adipogenic transcription factors, particularly SREBP1, promotes adipocyte hypertrophy, hyperplasia, increased visceral fat mass, and significant weight gain [7,8]. This adverse effect profile has severely restricted RSG’s clinical use despite its potent glucose-lowering effects. This underscores an urgent need for strategies that dissociate PPARγ’s metabolic benefits from its adipogenic effects [9]. Recent research continues to explore mechanisms of PPARγ’s divergent effects, highlighting the potential for selective PPARγ modulators (sPPARγMs) or adjunct therapies to achieve this uncoupling [10].

Natural products have emerged as a rich source of potential therapeutics and adjuncts for T2DM, offering multi-target effects often with improved safety profiles compared to synthetic drugs [11]. Among these, EMO—isolated from *Rhei Radix et Rhizoma* (Dahuang, *Rheum palmatum* L.), *Polygoni Cuspidati Rhizoma et Radix* (Huzhang, *Polygonum cuspidatum* Sieb. et Zucc.), and other traditional Chinese herbs—has shown direct effects on the PPARγ/SREBP1 axis and energy expenditure [12]. Historically valued for its anti-inflammatory and antimicrobial properties, extensive modern research has revealed EMO’s potent pharmacological activity against metabolic disorders, including obesity, hyperlipidemia, MASLD, T2DM, and its complications [13,14]. Crucially, EMO demonstrates direct effects relevant to the PPARγ/SREBP1 axis and energy expenditure. Studies show EMO can inhibit adipogenesis and adipocyte differentiation in WAT [15,16,17], promote WAT browning and thermogenesis via activation of pathways involving PPARγ coactivator 1α (PGC-1α) and uncoupling protein 1 (UCP1) [12,18], enhance peripheral glucose utilization, and improve hepatic and systemic insulin sensitivity [19]. These effects position EMO as a compelling candidate to potentially mitigate the adverse effects of PPARγ agonists like RSG while potentially enhancing their efficacy.

While other natural compounds like resveratrol [20] and puerarin [21] have shown some promise in reducing specific side effects of TZDs (e.g., bone loss or hepatotoxicity), no natural agent has been conclusively demonstrated to concurrently and effectively counteract the hallmark RSG-induced weight gain and adiposity while simultaneously preserving or enhancing its insulin-sensitizing effects through coordinated modulation of the distinct PPARγ downstream pathways driving adipogenesis (SREBP1) [16] and thermogenesis (PGC-1α/UCP1) [18]. Critically, no study has asked whether EMO can simultaneously block RSG-driven weight gain while preserving its glucose-lowering efficacy; addressing this gap could reposition a shelved drug.

Therefore, we hypothesize that EMO co-administration will mitigate RSG-induced weight gain, visceral adiposity, and associated metabolic dysfunction by uniquely modulating PPARγ activity: suppressing SREBP1-mediated lipogenesis while simultaneously augmenting PGC-1α-dependent thermogenesis. In the present study, using the leptin-deficient *ob*/*ob* mouse model of severe obesity and insulin resistance and in vitro 3T3-L1 adipocytes, we investigate the effects of EMO on RSG-induced metabolic changes. We specifically assess its ability to enhance RSG’s insulin-sensitizing properties while counteracting its pro-obesity effects. Utilizing RNA-sequencing and comprehensive molecular analyzes, we elucidate the key pathways involved, focusing on the SREBP1-regulated adipogenic program and the PPARγ/PGC-1α axis governing thermogenesis. Our findings may provide a clinically feasible route to restore RSG’s therapeutic utility in obesity-associated T2DM.

## 2. Results

### 2.1. EMO Enhances RSG-Induced Insulin Sensitization and Attenuates Pro-Obesity Effects

**Figure 1:**
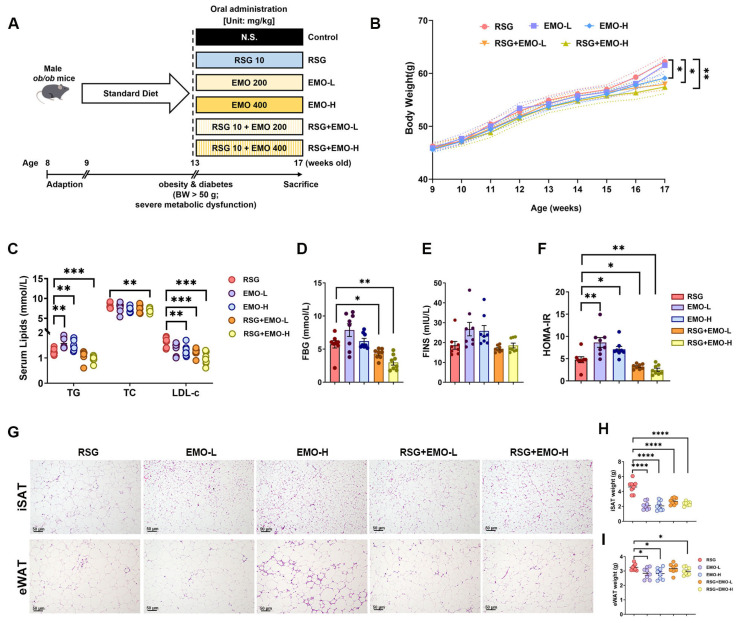
EMO improves insulin sensitivity and mitigates the pro-obesity effects of RSG in *ob*/*ob* mice. (**A**) Experimental design and treatment schedule. Eight-week-old male *ob*/*ob* mice were acclimated for one week, followed by a four-week period on a standard diet to induce obesity and diabetes. The mice were then treated daily for four weeks with either vehicle (Control), EMO-L (200 mg kg^−1^), EMO-H (400 mg kg^−1^), RSG (10 mg kg^−1^), or their combinations (RSG + EMO-L, RSG + EMO-H). (**B**) Changes in body weight over the treatment period. (**C**–**E**) Serum lipid profiles (TG, TC, and LDL-c), fasting blood glucose (FBG), and fasting insulin (FINS) levels. (**F**) Homeostatic Model Assessment for Insulin Resistance (HOMA-IR) scores calculated from FBG and FINS levels. (**G**,**H**) ISAT and eWAT weights. (**I**) Representative hematoxylin and eosin (H&E)-stained sections of iSAT and eWAT (200× magnification). Data are presented as mean ± SEM (*n* = 8 per group). * *p* < 0.05, ** *p* < 0.01, *** *p* < 0.001, and **** *p* < 0.0001 indicate statistical significance vs. indicated group.

The metabolic effects of EMO and RSG, administered either as monotherapies or in combination, were evaluated in *ob*/*ob* mice (Figure 1A). While EMO alone did not improve serum lipids compared with RSG monotherapy, the RSG + EMO-H combination significantly reduced TG, TC, and LDL-c levels (Figure 1C). As expected, RSG treatment improved insulin sensitivity, with RSG + EMO-H showing the most robust reductions in FBG and HOMA-IR (Figure 1D–F). RSG + EMO-L also enhanced glucose-lowering effects relative to RSG alone, indicating a potential dose-dependent enhancement of RSG efficacy (Figure 1D–F).

RSG monotherapy increased weight gain, adipocyte hypertrophy (in both iSAT and eWAT), and adipose mass (Figure 1B,G–I). These RSG-induced effects were attenuated by EMO co-treatment, particularly with EMO-H, which reduced iSAT mass and adipocyte size (Figure 1G–I). Both EMO monotherapy and its combination with RSG significantly ameliorated liver steatosis and renal dysfunction compared with RSG-treated groups, as shown by reduced serum markers of liver damage (ALT, AST) and renal impairment (CREA, UN), decreased hepatic lipid content, and improved liver weight parameters (Figure S1). Notably, EMO alone showed superior hepatoprotective effects compared with RSG combinations (Figure S1).

Safety assessments revealed that EMO (200 or 400 mg·kg^−1^·day^−1^) was well-tolerated. EMO-H monotherapy and RSG + EMO-H co-treatment maintained normal liver weights (*p* > 0.05, vs. controls) without inducing behavioral abnormalities. No treatment-related toxicity was observed, with normal organ-to-body weight ratios, and serum markers (TBIL, CREA, UN) across all EMO-treated groups (Figure S1).

These findings suggest that EMO enhances RSG’s insulin-sensitizing effects while mitigating its pro-obesity and metabolic complications in *ob*/*ob* mice, with no adverse effects at the tested doses.

### 2.2. EMO Modulates Adipogenesis and Thermogenesis Pathways in iSAT of RSG-Induced Obesity

**Figure 2:**
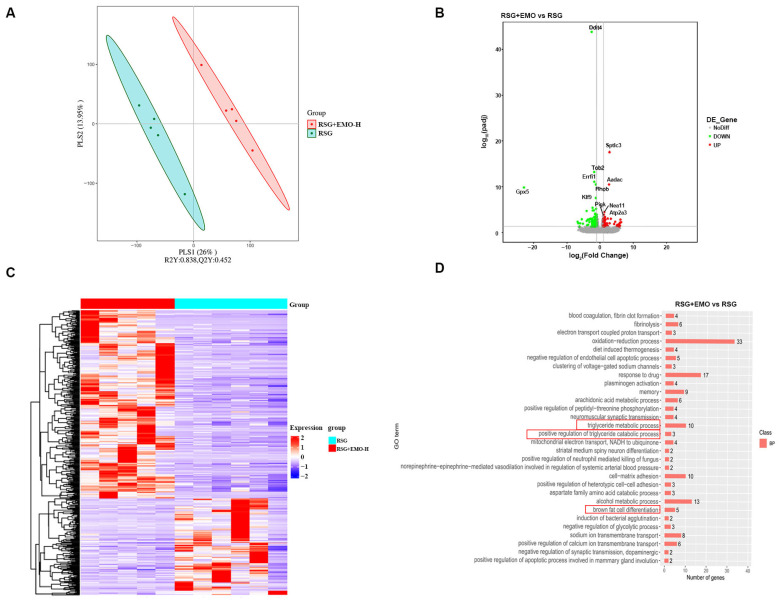
RNA-sequencing reveals modulation by EMO in RSG-induced obesity. (**A**) PLS-DA analysis indicates a distinct separation between the RSG and RSG + EMO-H groups within the iSAT of *ob*/*ob* mice. (**B**) A volcano plot displaying the DEGs between the groups. (**C**) A heatmap depicting hierarchical clustering of the DEGs. (**D**) GO enrichment analysis of the DEGs, emphasizing biological processes related to adipose tissue function; red boxes indicate GO terms related to triglyceride metabolic process, positive regulation of triglyceride catabolic process, and brown fat cell differentiation. *n* = 5 mice per group. Volcano plot highlights genes with |log2FC| > 1 and FDR < 0.05; upregulated genes in red, downregulated in green.

To elucidate the mechanisms underlying EMO’s attenuation of RSG-induced effects, we conducted RNA sequencing on iSAT, the depot most responsive to combination treatment. Differential expression analysis (FDR < 0.05, |log_2_FC| > 1) identified 462 differentially expressed genes (DEGs) between the RSG and RSG + EMO-H groups. Partial least-squares discriminant analysis (PLS-DA) showed clear separation of the treatment clusters (Figure 2A), confirming robust transcriptomic changes. Among these DEGs, 305 were up-regulated (e.g., *Sptlc3*, *Aadac*, *Atp2a3*) and 157 were down-regulated (e.g., *Tob2*, *Klf9*) in RSG + EMO-H verse RSG alone (Figure 2B,C). Pathway analysis revealed significant enrichment in triglyceride catabolism, calcium-ion transport, and adipocyte differentiation pathways (Figure 2D). Key findings included marked repression of the pro-adipogenic factor *Klf9* and strong induction of *Atp2a3*, a sarcoplasmic reticulum Ca^2+^-ATPase implicated in thermogenesis. These results suggest that EMO simultaneously suppresses lipogenic programs while activating energy-dissipating pathways within iSAT, helping to explain the observed reduction in adiposity.

### 2.3. EMO Inhibits RSG-Induced Adipogenesis via the SREBP1 Pathway in Ob/Ob Mice

**Figure 3:**
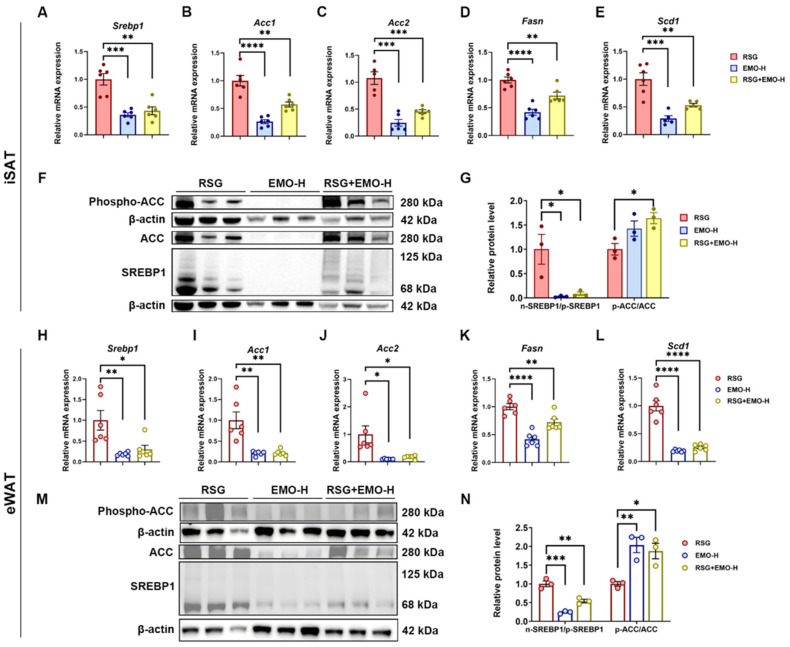
EMO inhibits RSG-induced adipogenesis via the SREBP1 pathway in *ob*/*ob* mice. (**A**–**E**) Relative mRNA levels of *Srebp1* and downstream adipogenic targets (*Acc1*, *Acc2*, *Fasn*, and *Scd1*) in iSAT. (**F**,**G**) Western blot analysis of SREBP1 and phosphorylated ACC (inactive form) in iSAT, with densitometric densitometry. (**H**–**L**) Relative mRNA expression of *Srebp1* and downstream adipogenic targets (*Acc1*, *Acc2*, *Fasn*, and *Scd1*) in eWAT. (**M**,**N**) Western blot analysis of SREBP1 and phosphorylated ACC in eWAT, with densitometry. All Western blots were performed in triplicate. Data are presented as mean ± SEM (*n* = 3–5 per group). * *p* < 0.05, ** *p* < 0.01, *** *p* < 0.001, and **** *p* < 0.0001 vs. indicated group.

Our RNA-seq analysis revealed that EMO + RSG modulates triglyceride metabolic pathways (Figure 2D). Further investigation identified SREBP1-mediated de novo lipogenesis (DNL) as a key regulatory mechanism. In both iSAT and eWAT, RSG treatment significantly increased mRNA levels of *Srebp1* and its downstream targets (*Acc1*, *Acc2*, *Fasn*, *Scd1*). Importantly, these effects were reversed by both EMO monotherapy and combination treatment (Figure 3A–E,H–L). Protein analysis revealed RSG promoted the mature (active) form of SREBP1, whereas EMO monotherapy suppressed SREBP1 maturation without influencing ACC phosphorylation. Remarkably, the combination treatment concurrently suppressed SREBP1 maturation and enhanced ACC phosphorylation (Figure 3F,G,M,N), suggesting a dual blockade of DNL. This inhibitory pattern exhibited depot specificity, with iSAT displaying the most pronounced effects.

Collectively, these data demonstrate that, while EMO alone downregulates lipogenic genes at the transcriptional level, its combination with RSG simultaneously disrupts both SREBP1 maturation and ACC phosphorylation, resulting in synergistic suppression of lipid accumulation that is most prominent in iSAT.

### 2.4. EMO Attenuates RSG-Induced Adipogenesis via the SREBP1 Pathway in 3T3-L1 Cells

**Figure 4:**
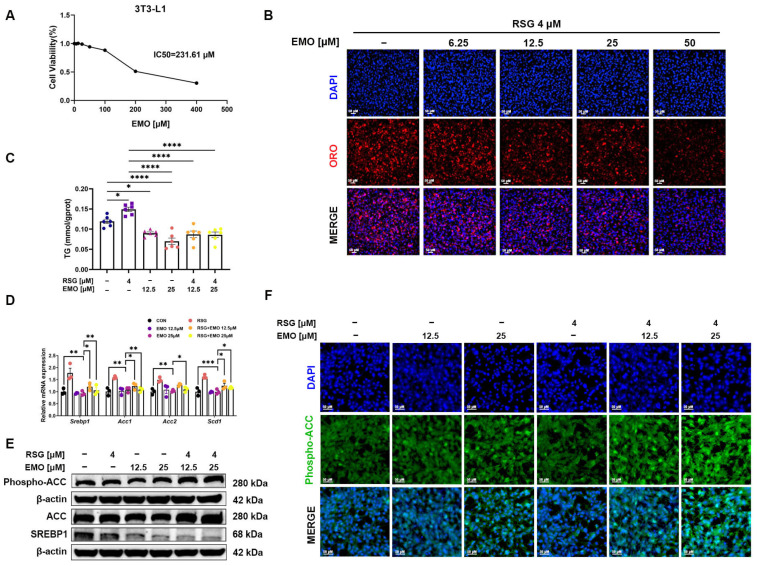
EMO attenuates RSG-induced adipogenesis in 3T3-L1 cells. (**A**) Cell-viability analysis of 3T3-L1 cells treated with EMO (6.25–500 μM). (**B**) ORO staining of 3T3-L1 cells treated with RSG (4 μM) and co-treated with EMO (6.25–50 μM) (200× magnification). (**C**) Quantification of intracellular TG content in 3T3-L1 cells treated with RSG (4 μM), EMO (12.5 or 25 μM), or co-treated with EMO. (**D**) Relative mRNA expression of *Srebp1* and downstream targets (*Acc1*, *Acc2*, and *Scd1*). (**E**) Western blot analysis of the SREBP1 and phosphorylated ACC. (**F**) Immunofluorescence images of phosphorylated ACC (200× magnification). All Western blots were performed in triplicate. Data are presented as mean ± SEM (*n* = 3–5 per group). * *p* < 0.05, ** *p* < 0.01, *** *p* < 0.001, and **** *p* < 0.0001 vs. indicated group.

To validate the in vivo anti-adipogenic effects of EMO, we examined its impact on RSG-treated 3T3-L1 adipocytes. Viability assays confirmed that EMO concentrations up to 100 μM were not cytotoxic (Figure 4A). RSG significantly increased lipid accumulation, as shown by intracellular TG levels and ORO staining compared with untreated controls (Figure 4B,C). EMO co-treatment (6.25 to 50 μM) dose-dependently attenuated RSG-induced lipid accumulation. In line with the in vivo data, RSG up-regulated *Srebp1* and its downstream targets (*Acc1*, *Acc2*, and *Scd1*), enhanced the mature SREBP1 protein and reduced ACC phosphorylation in 3T3-L1 cells (Figure 4D–F). EMO co-treatment (25 μM, showing maximal efficacy) reversed RSG-induced mRNA upregulation, suppressed mature SREBP1 protein, and dose-dependently increased ACC phosphorylation in 3T3-L1 cells (Figure 4D–F).

These in vitro findings support that EMO directly inhibits RSG-induced adipogenesis and SREBP1 pathway activation in 3T3-L1 adipocytes, thereby reinforcing the in vivo results.

### 2.5. EMO Enhances RSG-Mediated PPARγ Activation in 3T3-L1 Cells

**Figure 5:**
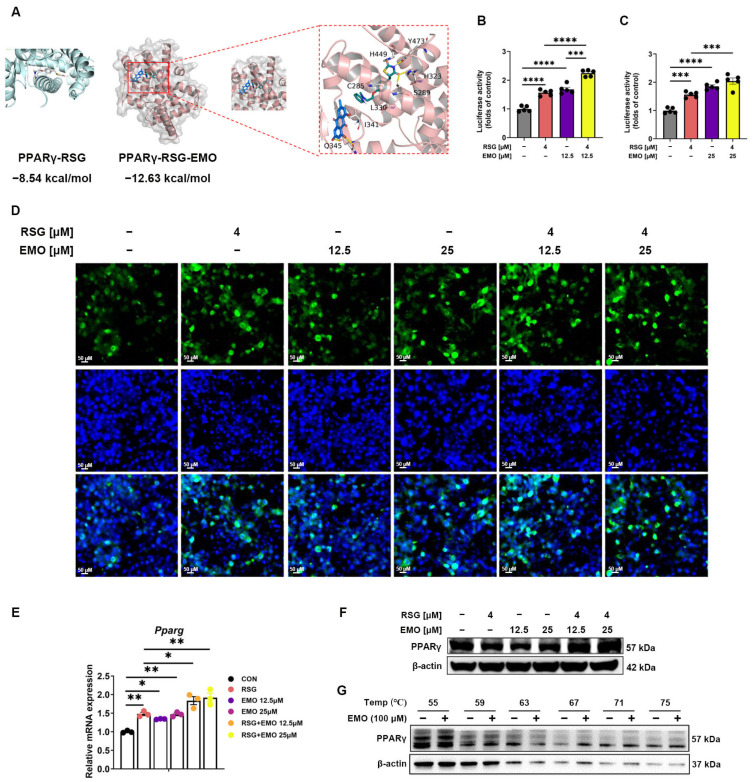
EMO enhances RSG-mediated PPARγ activation and transcriptional regulation in 3T3-L1 cells. (**A**) Molecular docking analysis indicates a putative binding mode of EMO adjacent to the RSG-PPARγ complex (ΔG = −8.54 kcal/mol) and the higher affinity of the ternary RSG-EMO-PPARγ complex (ΔG = −12.63 kcal/mol). The zoomed-in view highlights key residues involved in the interaction. (**B**,**C**) Luciferase reporter assay measuring PPARγ transactivation in 3T3-L1 cells treated with EMO (12.5 or 25 μM) and RSG (4 μM). (**D**) Immunofluorescence images of PPARγ nuclear translocation in 3T3-L1 cells treated with RSG (4 μM), EMO (12.5 or 25 μM), or their combination (200× magnification). (**E**) Relative mRNA levels of *Pparg*. (**F**) Western blot analysis of PPARγ protein. (**G**) CETSA assessing PPARγ thermal stability in 3T3-L1 cells treated with vehicle (1‰ DMSO) or EMO (100 μM, 1‰ DMSO). All Western blots were performed in triplicate. Data are presented as mean ± SEM (*n* = 3–5 per group). * *p* < 0.05, ** *p* < 0.01, *** *p* < 0.001, and **** *p* < 0.0001 vs. indicated group.

To investigate potential PPARγ modulation by EMO without exacerbating lipogenesis, we performed molecular docking analysis. The results indicated that EMO may occupy an auxiliary binding site adjacent to RSG on PPARγ (Figure 5A), though direct biophysical validation remains necessary.

Luciferase reporter assays showed that EMO alone induced modest PPARγ transactivation, while co-treatment with RSG further amplified transcriptional activity (Figure 5B,C). This effect was corroborated by immunofluorescence showing enhanced PPARγ nuclear translocation under co-treatment (Figure 5D). Notably, EMO upregulated *Pparg* mRNA without altering total PPARγ protein levels or thermal stability (Figure 5E–G).

These findings suggest that EMO potentiates RSG-mediated PPARγ activation while maintaining its suppressive effects on lipogenic signaling, supporting its role for EMO as a metabolic modulator rather than a full synergist.

### 2.6. EMO Amplifies RSG-Mediated Thermogenesis via the PPARγ-PGC-1α Axis in Adipose Tissues

**Figure 6:**
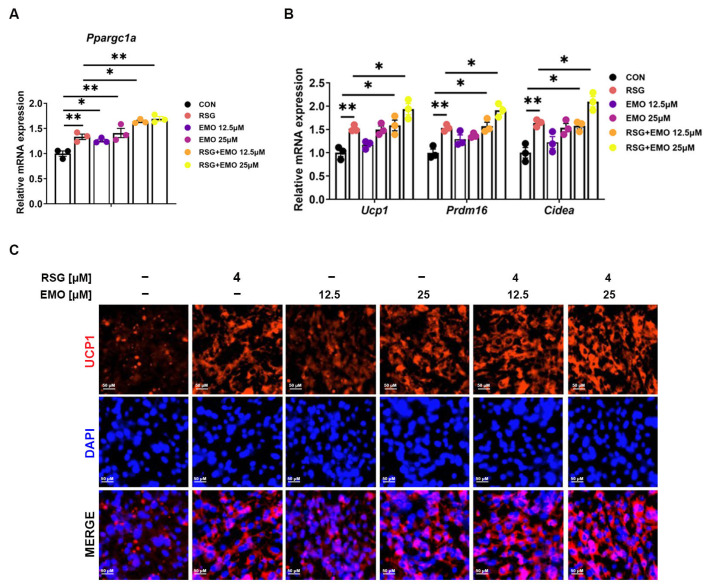
EMO modulates RSG-induced thermogenesis in 3T3-L1 cells via PPARγ-PGC-1α. (**A**) Relative mRNA levels of *Ppargc1a*. (**B**) Relative mRNA levels of thermogenic genes (*Ucp1*, *Prdm16*, and *Cidea*). (**C**) Immunofluorescence staining of UCP1 (200× magnification). Data are presented as mean ± SEM (*n* = 3 per group). * *p* < 0.05 and ** *p* < 0.01 vs. indicated group.

Having demonstrated that EMO synergistically enhances RSG-mediated PPARγ activation (Figure 5), we investigated potential cooperativity in PPARγ-driven thermogenic pathways. Focusing on the pivotal role of PPARγ-PGC-1α signaling in adipose browning, we first assessed its regulation in 3T3-L1 adipocytes. Both EMO monotherapy and co-treatment with RSG significantly increased *Ppargc1a* mRNA (Figure 6A) and coordinately upregulated thermogenic genes (*Ucp1*, *Prdm16*, *Cidea*; Figure 6B). Immunofluorescence analysis confirmed elevated UCP1 protein expression (Figure 6C), supporting EMO’s capacity to activate the PPARγ-PGC-1α-UCP1 axis independently of RSG.

**Figure 7:**
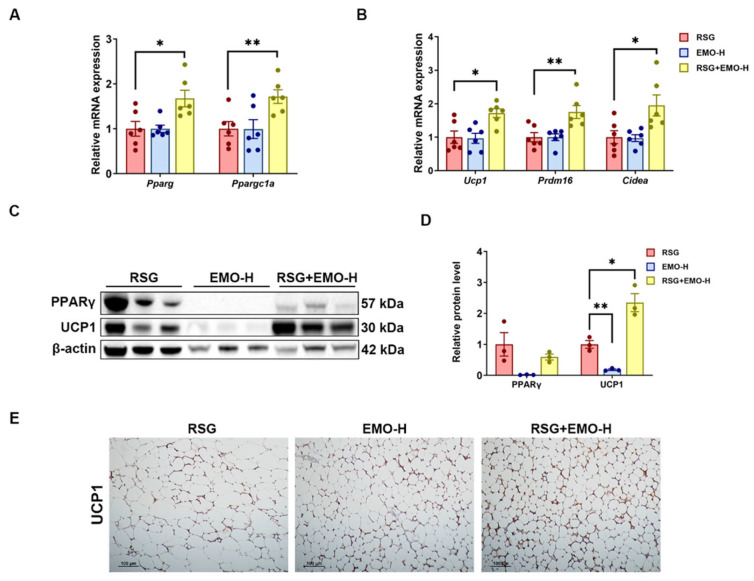
EMO modulates RSG-induced thermogenesis and browning in iSAT via the PPARγ-PGC-1α. (**A**,**B**) Relative mRNA levels of *Pparg*, *Ppargc1a*, and *thermogenic* genes (*Ucp1*, *Prdm16*, *Cidea*) in iSAT. (**C**,**D**) Western blot analysis of PPARγ and UCP1 proteins in iSAT, with densitometric quantification. (**E**) IHC staining of UCP1 in iSAT (200× magnification). All Western blots were performed in triplicate. Data are presented as mean ± SEM (*n* = 3–5 per group). * *p* < 0.05 and ** *p* < 0.01 vs. indicated group.

**Figure 8:**
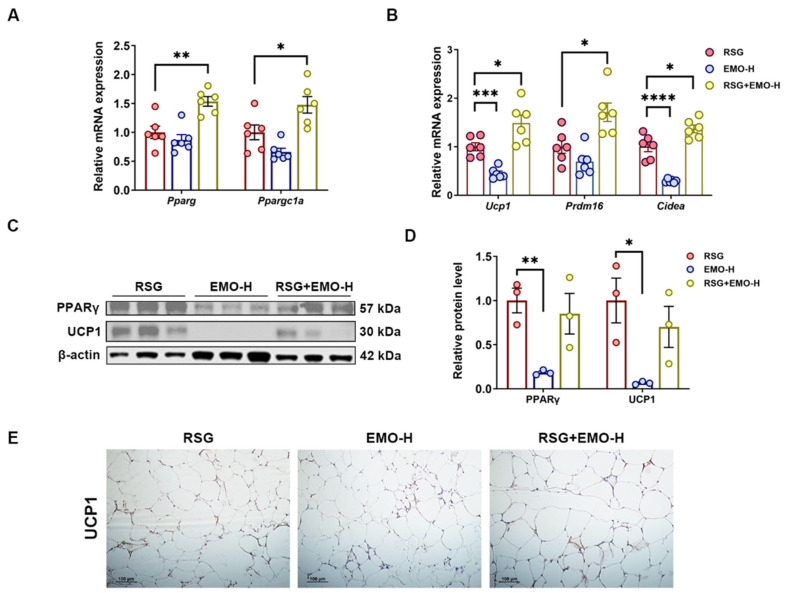
EMO modulates RSG-induced thermogenesis and browning in eWAT via the PPARγ-PGC-1α. (**A**,**B**) Relative mRNA levels of *Pparg*, *Ppargc1a*, and thermogenic genes (*Ucp1*, *Prdm16*, *Cidea*) in eWAT. (**C**,**D**) Western blot analysis of PPARγ and UCP1 proteins in eWAT, with densitometric quantification. (**E**) IHC staining of UCP in eWAT (200× magnification). All Western blots were performed in triplicate. Data are presented as mean ± SEM (*n* = 3–5 per group). * *p* < 0.05, ** *p* < 0.01, *** *p* < 0.001, and **** *p* < 0.0001 vs. indicated group.

In *ob*/*ob* mice, both EMO and RSG monotherapies upregulated thermogenic gene expression (*Ppargc1a*, *Ucp1*, *Prdm16*, *Cidea*) in iSAT and eWAT, with co-treatment producing synergistic effects (Figure 7A,B and Figure 8A,B). Notably, EMO alone reduced PPARγ protein levels in iSAT (Figure 7C,D), whereas co-treatment restored PPARγ abundance to RSG-alone levels, suggesting RSG-mediated PPARγ stabilization despite EMO’s transcriptional potentiation (Figure 5).

Importantly, UCP1 protein was markedly induced exclusively in iSAT (Figure 7C–E), aligning with its greater thermogenic potential compared with eWAT (Figure 8C–E). The depot-specific UCP1 induction in iSAT correlated with reduced adipocyte size (Figure 2), supporting a role for enhanced thermogenesis in the combinatorial therapy’s anti-obesity effects. Conversely, eWAT’s limited thermogenic response highlights the importance of targeting metabolically active depots.

These findings suggest that EMO and RSG jointly activate thermogenesis (PPARγ-PGC-1α-UCP1) while simultaneously inhibiting lipogenesis (SREBP1-ACC), providing a dual mechanism for enhanced metabolic benefits. The depot-specific UCP1 induction, with minimal expression in eWAT, further supports intrinsic differences in adipose thermogenic potential.

## 3. Discussion

PPARγ activation remains fundamental to T2DM management due to its robust insulin-sensitizing efficacy, yet its clinical utility is limited by adipogenic side effects. The thiazolidinedione RSG exemplifies this therapeutic dilemma: while effectively improving glycemic control, it stimulates adipocyte differentiation and lipid accumulation in iSAT [22], leading to weight gain and ectopic lipid deposition [23]. Although various natural compounds have demonstrated potential in mitigating drug-induced metabolic complications [20,21], our study supports EMO as a potential therapeutic adjunct that simultaneously enhances RSG’s insulin-sensitizing benefits while suppressing its adipogenic effects through three key mechanisms.

Through integrated investigation in both 3T3-L1 adipocytes and *ob*/*ob* mice, we show that EMO co-treatment synergistically enhances RSG’s glucose-lowering and insulin-sensitizing effects while effectively mitigating RSG-induced weight gain, adipocyte hypertrophy, and hepatic steatosis. Notably, this combinatorial therapy exhibits adipose depot selectivity. In iSAT, the EMO-RSG combination significantly upregulated thermogenic markers (*Ucp1*, *Ppargc1a*) and reduced adipocyte size, with markedly attenuated effects in eWAT (Figure 7 and Figure 8). This anatomical selectivity aligns with clinical evidence suggesting that browning of subcutaneous fat correlates with improved metabolic health [15,18], and provides a mechanistic basis for the observed attenuation of adipocyte hypertrophy, identifying iSAT as the primary target for the therapeutic approach.

The mechanistic basis of EMO’s ability to optimize RSG’s therapeutic index involves two complementary yet distinct pathways. First, EMO enhances PPARγ-mediated insulin sensitization, as suggested by in silico docking indicating a possible increase in the apparent binding affinity of RSG to PPARγ (Figure 5A), amplifying PPARγ transactivation (Figure 5B,C), and promoting its nuclear translocation (Figure 5D), without altering PPARγ protein stability (Figure 5E–G). This pattern is consistent with a cooperative-like modulation of PPARγ that may potentiate the transcription of genes involved in glucose metabolism. Second, and equally important, EMO potently suppresses RSG-induced lipogenesis and adipogenesis through inhibition of the SREBP1 pathway [16]. While both EMO monotherapy and combination therapy attenuated RSG-induced upregulation of *Srebp1* and its downstream targets *(Acc1*, *Acc2*, *Fasn*, *Scd1*) at the mRNA level in both iSAT and eWAT (Figure 3 and Figure 4), the combination therapy exerted a more profound inhibitory effect at the protein level, reducing the proteolytic maturation of SREBP1 and enhancing the phosphorylation (inactivation) of ACC (Figure 3F,G,M,N and Figure 4F). This dual inhibition of de novo lipogenesis was particularly evident in iSAT, correlating with the observed depot-specific reduction in lipid accumulation and induction of thermogenesis. These findings align with previous studies indicating that iSAT exhibits higher responsiveness to anti-lipogenic and thermogenic stimuli compared to visceral depots [24,25].

The requirement for higher EMO doses (200–400 mg kg^−1^ day^−1^) in the severely insulin-resistant *ob*/*ob* model aligns with previous reports in diet-induced obesity models [26]. Importantly, the 400 mg kg^−1^ day^−1^ dose provided optimal metabolic benefits (Figure 6, Figure 7 and Figure 8) while demonstrating excellent safety: (1) no alterations in liver weights or behavior; (2) improved serum markers (ALT, AST, TBIL) without affecting CREA/UN (Figure S1); and (3) maintained normal food intake and organ coefficients. These findings are supported by chronic toxicity studies in rodents [19], although careful dose translation remains necessary given interspecies metabolic differences.

**Figure 9:**
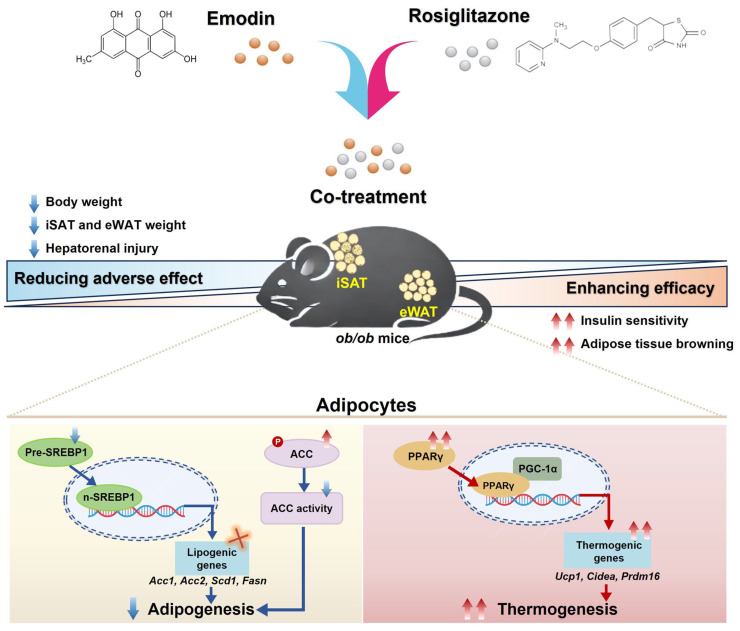
Proposed mechanism by which EMO may widen the therapeutic window of RSG. In *ob*/*ob* mice, EMO co-administration was associated with reduced body weight, adipose-tissue mass (iSAT and eWAT), and markers of hepatorenal injury, alongside improved glucose homeostasis and signs of adipose-tissue browning. Molecular analyzes suggest that EMO suppresses RSG-induced adipogenesis by attenuating SREBP1 maturation and down-regulating lipogenic genes (*Acc1*, *Acc2*, *Fasn*, *Scd1*), while concurrently enhancing PPARγ/PGC-1α-mediated thermogenesis via up-regulation of *Ucp1*, *Cidea*, *Prdm16*. These complementary actions could mitigate RSG’s adverse effects without overtly compromising its insulin-sensitizing benefits, providing a rationale for further exploration of dose-sparing combination therapy.

Our findings position the PPARγ–PGC-1α axis [27] as a central signaling hub (Figure 9), where EMO’s dual modulation establishes a positive feedback loop that enhances both insulin sensitivity and thermogenesis. This mechanism differs fundamentally from conventional TZD monotherapy and aligns with current efforts to develop tissue-selective PPARγ modulators [10,23]. The combination’s ability to maintain glycemic control while reducing adipogenic effects suggests potential for revitalizing RSG therapy, particularly if future studies confirm: (1) RSG dose-reduction feasibility [28]; (2) efficacy across sexes; and (3) applicability to patients with varying subcutaneous adipose mass.

Several limitations require consideration. First, using only male *ob*/*ob* mice [29] precludes sex-specific assessment and limits generalizability to polygenic obesity. Second, while demonstrating hepatorenal safety (Figure S1), this study lacked cardiovascular evaluations-crucial given TZDs’ historical associations. Third, the high EMO doses needed for maximal thermogenesis (400 mg kg^−1^ day^−1^) necessitate rigorous PK/PD validation before clinical translation. Future research should therefore prioritize: (1) inclusion of female and diet-induced models to address the first limitation; (2) comprehensive cardiovascular assessment to resolve the second concern; and (3) pharmacokinetic-guided dose optimization to overcome the third challenge.

## 4. Materials and Methods

### 4.1. Chemicals and Reagents

RSG (purity > 99%, Cat# T6772) was obtained from Topscience Co., Ltd. (Shanghai, China). EMO (purity ≥ 98%, Cat# S27967) was purchased from Shanghai Standard Technology Co., Ltd. (Shanghai, China). Oil Red O (Cat# O0625) was sourced from Sigma-Aldrich (St. Louis, MO, USA). Dimethyl sulfoxide (DMSO, Cat# D8418) and high-grade ethanol and isopropanol were also sourced from Sigma-Aldrich. Antibodies against SREBP-1 (sc-13551), PPARγ (sc-7273), PGC-1α (sc-518025), and UCP1 (sc-293418) were from Santa Cruz Biotechnology (Dallas, TX, USA); antibodies ACC (Cat# 3676) and phospho-ACC (Cat# 3661) were from Cell Signaling Technology (Danvers, MA, USA).

### 4.2. Animal Study Design

Animals and Ethics: Male C57BL/6J-*ob*/*ob* mice (8-week-old, 30–34 g; SLAC Laboratory Animal Co., Ltd., Shanghai, China), a model of leptin-deficient obesity and type 2 diabetes, were utilized. The mice were housed under specific pathogen-free (SPF) conditions (temperature of 22 ± 1 °C, relative humidity of 55 ± 5%, and a 12-h light/dark cycle) with unlimited access to standard rodent chow and water. After a one-week acclimatization period, the animals were randomly divided into five groups (*n* = 10 per group). All groups were given the standard diet ad libitum for 4 weeks to establish a model of naturally developed, severe metabolic disorder characterized by obesity and diabetes. Starting at 13 weeks of age, the groups received the following treatments daily for 4 consecutive weeks: The RSG group was administered RSG at a dose of 10 mg/kg/day [30]. EMO was given at two different doses: the EMO-L group received 200 mg/kg/day, and the EMO-H group received 400 mg/kg/day [26,31,32]. The RSG + EMO-L group received a co-administration of RSG (10 mg/kg/day) and EMO (200 mg/kg/day); the RSG + EMO-H group received a co-administration of RSG (10 mg/kg/day) and EMO (400 mg/kg/day).

Drug Administration and Sampling: All test compounds (RSG, EMO) and the vehicle (0.1% CMC-Na) were administered once daily via oral gavage at a volume of 10 mL/kg body weight. Body weight and fasting blood glucose (measured after a 6-h fast) were recorded weekly. At the end of the 8-week treatment period, mice were anesthetized with sodium pentobarbital (50 mg/kg, intraperitoneally). Blood was collected via cardiac puncture for serum isolation. iSAT, eWAT and liver were rapidly excised, weighed, and either fixed in 4% paraformaldehyde (PFA) for histological analysis or snap-frozen in liquid nitrogen for subsequent biochemical and molecular analyzes.

Ethical Approval: All experimental procedures involving animals were conducted in strict accordance with the National Institutes of Health (NIH) Guide for the Care and Use of Laboratory Animals and were approved by the Experimental Animal Ethics Committee of Shanghai University of Traditional Chinese medicine (Approval No.: PZSHUTCM211101028; Approval Date: 15 November 2021).

### 4.3. Biochemical Analysis

Serum triglycerides (TG), total cholesterol (TC), high-density lipoprotein cholesterol (HDL-c), and low-density lipoprotein cholesterol (LDL-c), alanine aminotransferase (ALT), aspartate aminotransferase (AST), total bilirubin (TBIL), total bile acid (TBA), fasting blood glucose (FBG), creatinine (CREA), and urea nitrogen (UN) were quantified using an automated biochemical analyzer (TBA-40FR, Toshiba, Tokyo, Japan). Serum insulin was determined using mouse ELISA kits from Shanghai Enzyme-linked Biotech Co., Ltd. (Catalog No. ml001983, Shanghai, China). Hepatic lipids were extracted from 100 mg of liver tissue homogenized in ethanol, with TG/TC measured using enzymatic kits (Catalog Nos. A110-1-1, A111-1-1, Nanjing Jiancheng Bioengineering Institute, Nanjing, China).

### 4.4. Histopathology and Immunohistochemistry

Tissue processing was performed as previously described [33]. Briefly, tissues (liver, iSAT, eWAT) were fixed in 4% PFA, paraffin-embedded, and sectioned at a thickness of 5 μm. Hematoxylin and eosin (H&E) staining (Cat# BA-4041, BA-4042; Baso, Wuhan, China) was performed using standard protocols. For lipid visualization, frozen liver sections were stained with Oil Red O at a thickness of 8 μm.

Immunohistochemistry (IHC): Deparaffinized sections underwent antigen retrieval in a 10 mM citrate buffer at pH 6.0, were blocked with 5% BSA, and incubated with an anti-UCP1 antibody (1:200; Cat# 72298S, Cell Signaling Technology) overnight at 4 °C. An HRP-conjugated secondary antibody (1:1000; Cat# SA1022; Bosterbio, Wuhan, China) was applied for 1 h, followed by DAB development. Images were acquired at 200× magnification (Axiolab5, ZEISS, Oberkochen, Germany).

Cell staining: Differentiated 3T3-L1 adipocytes were fixed in 4% PFA, stained with Lipid-Red (Cat #LD03; Dojindo, Hamamatsu, Japan), and counterstained with DAPI (Cat# C1002; Beyotime, Beijing, China). Fluorescence images were captured at 200× magnification (ImageXpress^®^ Micro 4, Molecular Devices, San Jose, CA, USA).

### 4.5. 3T3-L1 Adipocyte Differentiation

3T3-L1 preadipocytes (Cat# GNM25; Cell Bank of Chinese Academy of Sciences, Shanghai, China) were cultured in DMEM (Cat# 11965092; Gibco, Grand Island, NY, USA) with 10% FBS (Cat# SH30084.03; HyClone, Logan, UT, USA) and 1% penicillin/streptomycin (Cat# 15140122; Gibco). At 2 days post-confluence, differentiation was induced: Day 0–2: Differentiation medium I (DMEM/10% FBS, 0.5 mM IBMX, 1 μM dexamethasone, 1 μg/mL insulin); Day 2–8: Differentiation medium II (DMEM/10% FBS, 1 μg/mL insulin) [34]. The media were refreshed every 48 h. Compounds—EMO at concentrations of 12.5 or 25 μM and RSG at 4 μM—or vehicle (0.1% DMSO) were added during the differentiation process. EMO and RSG were dissolved in DMSO and then incorporated into the differentiation medium at their respective final concentrations: 12.5 or 25 μM for EMO and 4 μM for RSG.

### 4.6. Cell Viability Assay

3T3-L1 cells (5 × 10^3^/well) were seeded in 96-well plates and treated with EMO at concentrations ranging from 6.25 to 400 μM for 24 h. Subsequently, CCK-8 reagent (10 μL/well; Cat# C0046, Beyotime) was added, followed by a 2-h incubation period. The absorbance was then measured at 450 nm using an ImageXpress^®^ Micro 4 instrument from Molecular Devices.

### 4.7. Quantitative Real-Time PCR (qPCR)

Total RNA was isolated from snap-frozen adipose tissue or cultured cells using TRIzol (Thermo Fisher Scientific, Waltham, MA, USA) and quantified with a NanoDrop 2000c (NanoDrop Technology, Wilmington, DE, USA). This RNA was then reverse transcribed into cDNA and analyzed via Real-Time PCR on a StepOne PCR system (Applied Biosystems, Foster City, CA, USA), employing PowerUp SYBR Green Master Mix (Thermo Fisher Scientific, Waltham, MA, USA). Target gene expression levels were normalized against actin, and the analysis was conducted using the 2^−ΔΔT^ method. Primer sequences are provided in Supplementary Table S1.

### 4.8. Western Blotting

Tissues or cells were lysed in RIPA buffer (Cat# P0013B; Beyotime) supplemented with protease and phosphatase inhibitors (Cat# 4906837001, 4693132001; Roche, Roche Diagnostics, Indianapolis, IN, USA) for 30 min on ice, followed by centrifugation at 12,000× *g* for 15 min at 4 °C. Proteins concentrations were determined using a BCA assay kit (Cat# P0012S; Beyotime). Equal amounts of protein (30 μg per lane) were separated by 10% SDS-PAGE using a Mini-Protean^®^ Tetra System (Bio-Rad, Hercules, CA, USA) at 60 V constant voltage. Subsequently, proteins were electrophoretically transferred to PVDF membranes (Cat# IPFL00010; Millipore, Burlington, MA, USA) using a rapid transfer system (Genscript, Piscataway, NJ, USA) for 10 min. Membranes were then blocked with 5% non-fat milk in TBST for 1 h at room temperature [35]. Primary antibodies (listed in Supplementary Table S2) were incubated overnight at 4 °C (diluted 1:1000), followed by incubation with HRP-conjugated secondary antibodies (diluted 1:5000; Cat# 7074S/7076S, CST, Danvers, MA, USA) for 1 h at room temperature. Protein bands were visualized using Omni-ECL™ Femto substrate (Epizyme, Cambridge, MA, USA) and imaged with a Tanon-5200 imaging workstation (Tanon Science & Technology Co., Ltd., Shanghai, China). For quantitative analysis, band intensities were measured using ImageJ software (NIH, Bethesda, MD, USA). All target protein levels were normalized to β-actin (Cat# ET1702-67; Huabio, Hangzhou, Zhejiang, China) as internal control. Statistical analysis was performed on at least three independent biological replicates. Data are presented as mean ± SEM of the relative protein expression (target protein/β-actin ratio).

### 4.9. Luciferase Reporter Assay

PPARγ transcriptional activity was assessed using HEK293-PPARγ-luc cells, which were kindly provided by Prof. Guangbo Ge from Shanghai University of Traditional Chinese Medicine, China. The assessment was conducted as previously described [36]. Briefly, cells were seeded in a 96-well plate at a density of 5 × 10^3^ cells per well. After a 24-h incubation period, the cells were treated for 24 h with DMEM (vehicle control), 4 μM RSG (positive control), EMO at concentrations of either 12.5 or 25 μM, or combination treatments (EMO at 12.5/25 μM + 4 μM RSG). Luciferase activity was measured using the Steady-Glo^®^ Luciferase Assay System (Cat# E2510, Promega, Madison, WI, USA), following the manufacturer’s instructions. Luminescence was then quantified using a Synergy H4 microplate reader (BioTek Instruments, Winooski, VT, USA).

### 4.10. RNA Sequencing Analysis

Total RNA was extracted from iSAT samples (*n* = 5 per group) utilizing the TRNzol Universal Reagent (Tiangen, Beijing, China) [35]. The concentration, purity, and integrity were determined using a NanoDrop 2000 spectrophotometer (Thermo Fisher Scientific, USA) and an Agilent 2100 Bioanalyzer with the RNA 6000 Nano assay kit (Agilent, Santa Clara, CA, USA). RNA-seq libraries were constructed using the TIANSeq Fast RNA Library kit (Illumina, San Diego, CA, USA), following enrichment with the TIANSeq mRNA Capture Kit (Tiangen, China). After adaptor trimming and quality filtering with Trimmomatic, clean reads were assessed with FastQC and then aligned to the Mus musculus GRCm38/mm10 reference genome using STAR (v2.7.10). The mean sequencing depth was ~30 million paired-end reads per sample. Differential expression was analyzed using DESeq2 (v1.34.0). Genes with an absolute fold change ≥ 2 (|log_2_FC| ≥ 1) and a false discovery rate (FDR)–adjusted *p*-value < 0.05 were considered significantly differentially expressed. The potential roles of DEGs were elucidated using GO functional enrichment and KEGG pathway analysis, performed via topGO (v2.26.0) and clusterProfiler (v3.12.0). A GO term or metabolic pathway was considered significantly enriched for DEGs when the false discovery rate (FDR)-adjusted *p*-value was less than 0.05.

### 4.11. Immunofluorescence Staining

The 3T3-L1 cells were fixed with 4% paraformaldehyde (PFA) for 15 min at room temperature, permeabilized using 0.5% Triton X-100 (Sigma-Aldrich, St. Louis, MO, USA) in phosphate-buffered saline (PBS) for 10 min, and then washed three times with PBS. To block non-specific binding, the cells were incubated with 5% bovine serum albumin (BSA) in PBS for 1 h at room temperature. Subsequently, the cells were incubated overnight at 4 °C with primary antibodies (1:200 dilution; Cat# sc-7273, Santa Cruz, Dallas, TX, USA). After washing with PBS, the cells were incubated for 1 h at room temperature with a fluorophore-conjugated secondary antibody (1:1000 dilution; Cat# A-11008, Invitrogen, Carlsbad, CA, USA) and a DAPI solution. Following additional PBS washes, imaging and analysis were performed using an ImageXpress^®^ Micro 4 system from Molecular Devices [33].

### 4.12. Molecular Docking

The binding mode of EMO to the peroxisome proliferator-activated receptor gamma (PPARγ) ligand-binding domain (LBD) was investigated through molecular docking. The crystal structure of human PPARγ LBD (PDB ID: 5YCP) [37] was prepared using AutoDockTools 1.5.7, which involved the removal of water molecules and co-crystallized ligands, and the addition of polar hydrogens. The 3D structure of EMO (PubChem CID: 3220) was energy-minimized using the MMFF94 force field. The docking simulations were conducted using AutoDock Vina 1.2.3 [38].

### 4.13. Statistical Analysis

Quantitative data are expressed as mean ± standard error of the mean (SEM). Statistically significant differences were assessed using a two-tailed Student’s *t*-test or one-way analysis of variance (ANOVA). GraphPad Prism 8.0.1 statistical software (GraphPad Software, San Diego, CA, USA) was used for statistical analysis. Values of *p* < 0.05 were considered statistically significant [33].

## 5. Conclusions

In summary, our study demonstrates that EMO serves as a highly effective adjunctive therapy to RSG by reprogramming PPARγ downstream pathways. Specifically, it enhances the insulin-sensitizing and glucose-lowering effects of RSG through cooperative PPARγ activation while concurrently inhibiting SREBP1-mediated adipogenesis and promoting PGC-1α-driven thermogenesis via spatially restricted modulation of PPARγ effectors in subcutaneous adipose tissue. These complementary actions collectively mitigate RSG-induced weight gain and visceral adiposity, thereby redefining the therapeutic index of thiazolidinediones. Our findings underscore the potential of emodin as a naturally sourced combination partner for PPARγ agonists, offering a clinically actionable strategy to overcome weight gain-associated discontinuation in the pharmacotherapy of type 2 diabetes with obesity comorbidity.

## Data Availability

The data generated during this study are available if the requests are reasonable due to the confidentiality of the data.

## Supplementary Materials

The following supporting information can be downloaded at: https://www.mdpi.com/article/10.3390/ph18121810/s1, Figure S1: EMO alleviates RSG-induced liver steatosis and hepatorenal dysfunction in *ob*/*ob* mice; Table S1: Primer sequences for RT-qPCR; Table S2: Antibodies information.

## Abbreviations

The following abbreviations are used in this manuscript:

ACC	acetyl-CoA carboxylase
ALT	alanine aminotransferase
AST	aspartate aminotransferase
BAT	brown adipose tissue
CCK-8	cell counting kit-8
CIDEA	cell death-inducing DNA fragmentation factor-like effector A
CMC-Na	sodium carboxy methylcellulose
CREA	creatinine
DEGs	differentially expressed genes
DMSO	dimethyl sulfoxide
EMO	emodin
eWAT	epididymal white adipose tissue
FASN	fatty acid synthase
FBG	fasting blood glucose
FBS	fetal bovine serum
FC	fold change
FINS	fasting insulin
H&E	hematoxylin-eosin
HDL-c	high-density lipoprotein cholesterol
IBMX	3-isobutyl-1-methylxanthine
IHC	immunohistochemistry
iSAT	interscapular subcutaneous adipose tissue
LDL-c	low-density lipoprotein cholesterol
MASLD	metabolic dysfunction-associated steatotic liver disease
ORO	oil red o
PPARγ	peroxisome proliferator-activated receptor γ
PGC-1α	peroxisome proliferator-activated receptor-γ coactivator 1α
PRDM16	PR domain containing 16
P/S	penicillin/streptomycin
RSG	rosiglitazone
SCD1	stearoyl-CoA desaturase-1
SREBP1	sterol regulatory element-binding protein 1
T2DM	type 2 diabetes mellitus
TC	total cholesterol
TZD	thiazolidinedione
TBIL	total bilirubin
TG	triglyceride
TBA	total bile acid
UCP1	uncoupling protein 1
UN	urea nitrogen
WAT	white adipose tissue
