# Supplementary material for: Emodin Enhances Rosiglitazone’s Therapeutic Profile by Dual Modulation of SREBP1-Mediated Adipogenesis and PPARγ-Driven Thermogenesis
Source: Pharmaceuticals (Basel). 2025 Nov 27;18(12):1810. doi: 10.3390/ph18121810 (PMC12736021; doi:10.3390/ph18121810)
Supplement: Supplementary file 1 [file pharmaceuticals-18-01810-s001.zip › pharmaceuticals-4005739-supplementary.pdf]

Emodin Enhances Rosiglitazone's Therapeutic Profile by Dual Modulation of SREBP1-Mediated Adipogenesis and PPARγ-Driven Thermogenesis

Meng Li[1,2,6†], Yi-Rong Wang[1,2†], Xue Wang[1,5], Xiao-Li Xiao[1], Yun-Hong Sun[1,2,4], Sheng-An Zhang[1,2], Yan-Qi Dang[1,2], Kai Wang[3], Wen-Jun Zhou[1,2,*]

1 Institute of Digestive Diseases, Shanghai University of Traditional Chinese medicine, Shanghai 200032, China;

2 State Key Laboratory of Integration and Innovation of Classical Formula and Modern Chinese Medicine, Shanghai 201203, China;

3 Experiment Center for Science and Technology, Shanghai University of Traditional Chinese Medicine, Shanghai 201203, China;

4 School of Public Health, Shanghai University of Traditional Chinese Medicine, Shanghai 201203, China;

5 CAS Engineering Laboratory for Nutrition, Shanghai Institute of Nutrition and Health, University of Chinese Academy of Sciences, Chinese Academy of Sciences, Shanghai 200031, China;

6 School of Biological Sciences, Nanyang Technological University, Singapore 637551, Singapore.

***** Correspondence: zhouwenjun@shutcm.edu.cn, wjzhou678@163.com, 0000-0003-4167-0337.

† Meng Li, Yi-Rong Wang have contributed equally to this work.

Supplementary Figure

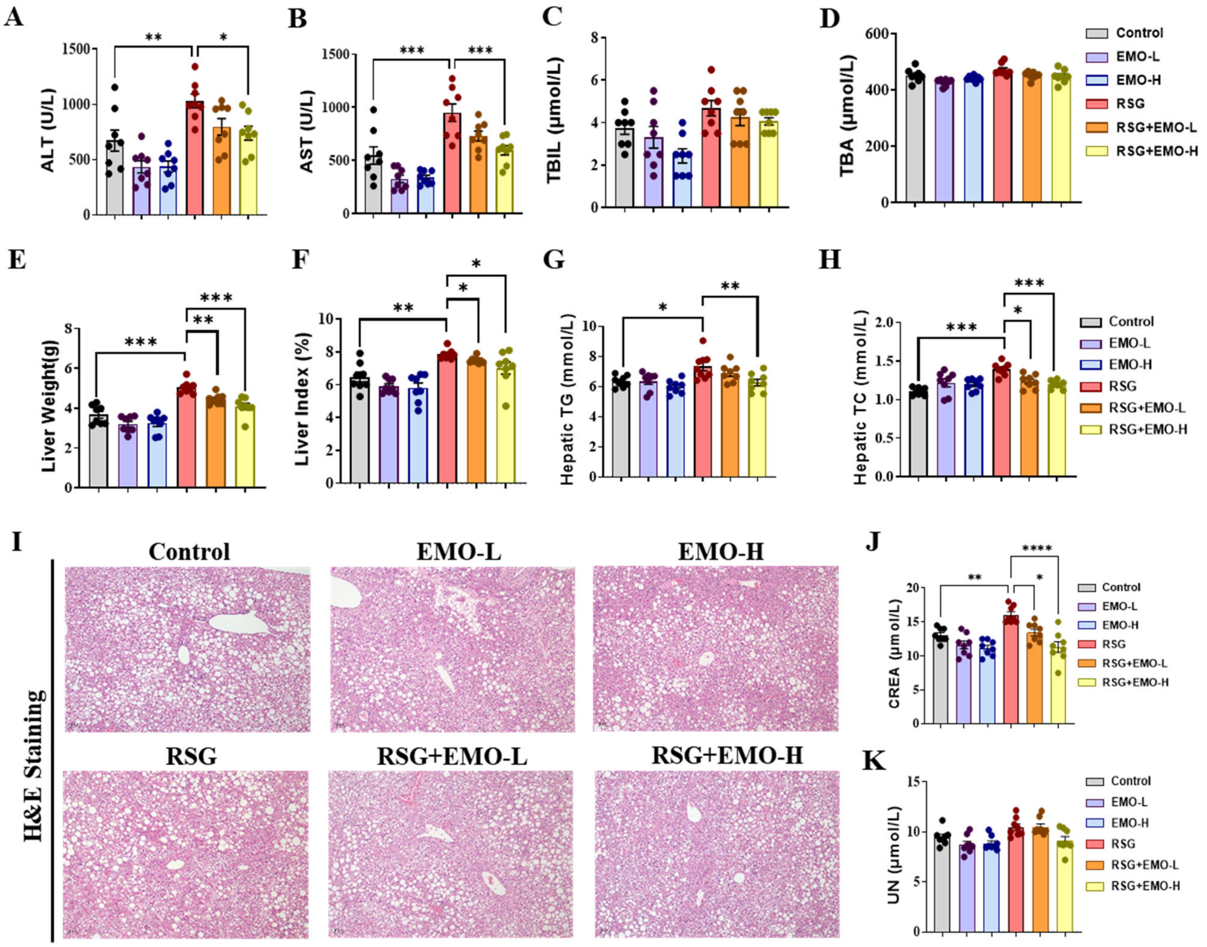

Figure S1: EMO alleviates RSG-induced liver steatosis and hepatorenal dysfunction in *ob/ob* mice. **A-D:** Serum levels of ALT, AST, TBIL, and TBA. **E, F:** Liver weight and liver index (ratio of liver weight to body weight). **G, H:** Liver TG and TC content. **I:** Representative H&E staining of liver sections (magnification 200×). **J, K:** Serum levels of CREA and UN. Data are presented as mean ± SEM (n = 8 per group). $^{*}P < 0.05$, $^{**}P < 0.01$, $^{***}P < 0.001$, and $^{****}P < 0.0001$ indicate significance between groups.

Supplementary Tables

Supplementary Table S1. Primer sequences for RT-qPCR

Primers	Forward	Reverse
Actin	GAGACCTTCAACACCCCAGC	ATGTCACGCACGATTTCCC
Srebp1	TCTGGAGACATCGCAAACAAG	GGATGAGGTTCCAAAGCAGAC
Acc1	CCACAGAACTTACAAGGCACG	GAATTGTGAGGGTCGGCG
Acc2	TCCCCGAAGACGTTTACGA	CTCTCAGCCTTGCGGATACC
Fasn	GTCAGATCTGCTGTTGAGCACAG	AATGATGCCGTCAGGTTTCAG
Scd1	ATTGCCTATGAACTCAACAGCG	TGCCATAGTGGTTGAGGTTGG
Pparg	CGTAGAAGCCGTGCAAGAGA	CATCTTTATTCATCAGGGAGGC
Ppaggc1a	TGGCACGCAGCCCTATTC	GAGGATCTACTGCCTGGGGAC
Ucp1	CGGCTTAATGACTGGAGGTG	GCATTGTAGGTCCCCGTGTAG
Prdm16	CACTCCCTCTACCCCTTTACG	CGGGTTTGGCCTCTTTTG
Cidea	CTTCCTCGGCTGTCTCAATG	GAACTGTCCCGTCATCTGTGC

Supplementary Table S2. Antibodies information. Abbreviations: WB, western blot; IF, immunofluorescence; IHC, immunohistochemistry

Antibody	Catalog number	Company	Use
**SREBP-1 (2A4) Mouse mAb**	sc-13551	Santa Cruz Biotechnology	WB
**ACC (C83B10) Rabbit mAb**	3676S	Santa Cruz Biotechnology	WB and IF
**Phospho-ACC (Ser79) (D7D11) Rabbit mAb**	11818S	Cell Signaling Technology	WB
**PPAR gamma Mouse antibody**	bsm-33436M	BIOSS	WB and IF
**UCP1 (E9Z2V) XP ® Rabbit mAb**	72298S	Cell Signaling Technology	WB, IF, and IHC
**Anti-beta Actin (HRP conjugated)**	ET1702-67	HuaBio	WB
**Anti-rabbit IgG, HRP-linked Antibody**	7074	Cell Signaling Technology	WB
**Anti-mouse IgG, HRP-linked Antibody**	7076	Cell Signaling Technology	WB
**Alexa Fluor goat anti-rabbit 488**	A-11034	Thermo Fisher Scientific	IF
**Alexa Fluor goat anti-rabbit 647**	A-21245	Thermo Fisher Scientific	IF
